# Yellow Fever

**Published:** 1902-04

**Authors:** 

**Affiliations:** Havana


### Abstracts and Selections.

### Yellow Fever.

(Translated for the Texas Medical Journal.)

Our contemporary, the *Havana Sun,* published recently the following humorous article, which we reproduce, *verbatim,* so that Dr. Gorgas may have the satisfaction of having a flea (bug) put in his right ear and then another one in his left ear. He praises the extinction of yellow fever in this fortunate island in two languages, and gives thanks to God.—*El Nuevo Pais.*

"A FLEA IN THEIR EARS."

We cannot quit thinking in these moments, of our great poet Henry W. Longfellow, and the estimation in which he held mosquitos, after that which has just occurred here, in the recent sessions of the Sanitary Congress, miscalled International, which just closed last week in the city of Havana.

"And around him the mosquito (Stegomyia)
Sang his war-song."

(Hiawatha, page 9, verse 13.)

The report of Dr. William C. Gorgas, Chief of the Sanitary Department of Havana, for the month of December of the year just gone by, says there were formed two "brigades" for the extermination of mosquitos. One of these was designated, the Stegomyia brigade, and the other the Anopheles brigade. This report says, that during the month of December 16,121 houses were inspected and oiled by the Stegomyia brigade; but it does not specify how many thousand more houses were inspected and oiled by the other brigade, the corps of the army of the Anopheles, which ought to consist, we suppose, of two or more brigades, of some species, more or less genuine, of the general brigade. It is probable that our readers wish to know how this war and destructive campaign on mosquitos is conducted by these raw sanitary recruits inside the houses in this city. To satisfy their natural curiosity, we will say that these *chassuers* are armed with a small oil can, squirt-can, filled with petroleum, and having been trained to manage them quickly and with dexterity (according to the true type of French soldiers of this name) with much skill and exactness of aim this light infantry discharges its destructive arm, and from the narrow mouth of the squirt-can, flies a small quantity of oily liquid, into cisterns, sinks, drain-pipes, etc. So marching along resolutely from house to house. Millions of the larvæ of the enemy are in this way daily exterminated, to escape from which fate, these diminutive musical creatures continue on the wing flying from one place to another, procreating their species with equal rapidity as before, apparently in burlesque, singing their plaintive song just as if nothing at all had happened to them.

In Mantanzas, Cárdenas, Sagua, Caibarién, Nuevitas, Gibara, Baracoa, Guantánimo, Santiago de Cuba, Manzanillo, Cienfuegos, Batabanó, and all of the other sea-coast cities of Cuba, even the other side of the bay of Havana, in Regla, Casa Blanca, in all of which places, they have not put in practice any measure of exterminating mosquitos whatever, yet nevertheless yellow fever has disappeared there just as in Havana. "How is it then?" asks Dr. A. M. Fernandez Ibarra (a physician of New York) of Dr. Gorgas, in the discussion in which both took part, on this subject in the Sanitary Congress, "that exactly the same good results followed, killing the mosquitos, and in letting them live tranquilly?"

We venture to follow the military Dr. Gorgas, favoring with all the logical arguments that opposed the mild Cuban, Dr. Fernandez Ibarra, that to form other powerful brigades of *chassuers* to exterminate bed-bugs and fleas (these blood-sucking insects have been lately proven the cause of the transmission of the bubonic plague, and intermittent fever, from one person to another). Therefore, then may it not be that they also transmit the yellow fever?

Mosquitos unquestionably transmit black vomit and paludial fevers, but are in no conception the *only means* of infection of these diseases.

This is a flea (bug) that we greatly regret to have to put in the ear (according to the English proverb) of the Chief of the Sanitary Department of Havana, and of all of the members of the Sanitary Congress who without thinking approved of the theory of that the mosquito *Stegomyia fasciata* is the only means proven until now of transmitting yellow fever. It is not only the only means of transmission, but even this subject has not been verified.

FREISCHUTZ.

Havana, Feb. 26th.

